# Noncutaneous malignant melanoma: a prognostic model from a retrospective multicenter study

**DOI:** 10.1186/1471-2407-10-167

**Published:** 2010-04-28

**Authors:** Hyo Song Kim, Eun Kyoung Kim, Hyun Jung Jun, Sung Yong Oh, Keon Woo Park, Do Hyoung Lim, Soon Il Lee, Jung Han Kim, Kyoung Mee Kim, Dae Ho Lee, Jeeyun Lee

**Affiliations:** 1 Division of Hematology-Oncology, Department of Medicine, Samsung Medical Center Sungkyunkwan University School of Medicine, Seoul, Korea; 2 Department of Oncology, University of Ulsan College of Medicine, Asan Medical Center, Seoul, Korea; 3 Department of Internal Medicine, Dong-A Cancer Center, Dong-A University College of Medicine, Busan, Korea; 4 Department of Hematology-Oncology, Dankook University School of Medicine, Cheonan, Korea; 5 Department of Surgery, Samsung Medical Center Sungkyunkwan University School of Medicine, Seoul, Korea; 6 Division of Medical Oncology, Yonsei Cancer Center, Yonsei University College of Medicine, Seoul, Korea; 7 Department of Pathology, Samsung Medical Center Sungkyunkwan University School of Medicine, Seoul, Korea

## Abstract

**Background:**

We performed multicenter study to define clinical characteristics of noncutaneous melanomas and to establish prognostic factors patients who received curative resection.

**Methods:**

Of the 141 patients who were diagnosed of non-cutaneous melanoma at 4 institutions in Korea between June 1992 and May 2005, 129 (91.5%) satisfied the selection criteria.

**Results:**

Of the 129 noncutaneous melanoma patients, 14 patients had ocular melanoma and 115 patients had mucosal melanoma. For mucosal melanoma, anorectum was the most common anatomic site (n = 39, 30.2%) which was followed by nasal cavity (n = 30, 23.3%), genitourinary (n = 21, 16.3%), oral cavity (n = 14, 10.9%), upper gastrointestinal tract (n = 6, 4.7%) and maxillary sinus (n = 5, 3.9%) in the order of frequency. With the median 64.5 (range 4.3-213.0) months follow-up, the median overall survival were 24.4 months (95% CI 13.2-35.5) for all patients, and 34.6 (95% CI 24.5-44.7) months for curatively resected mucosal melanoma patients. Adverse prognostic factors of survival for 87 curatively resected mucosal melanoma patients were complete resection (R1 resection margin), and age > 50 years. For 14 ocular melanoma, Survival outcome was much better than mucosal melanoma with 73.3% of 2 year OS and 51.2 months of median OS (*P *= .04).

**Conclusion:**

Prognosis differed according to primary sites of noncutaneous melanoma. Based on our study, noncutaneous melanoma patients should be treated differently to improve survival outcome.

## Background

Melonoma is originated from melanocytes, located in skin epidermis, eye, and epithelia of the nasal cavity, oropharynx, anus, and genitourinary tract. Melanoma comprises about 4.3% of all new cancer diagnosed in United States according to the National Center for Health Statistics database report[1]. Compared to the cutaneous melanoma which consists of majority of disease, noncutaneous melanomas are relatively rare, which are comprised of ocular and various mucosal sites such as anorectal, vaginal, nasal, and gastrointestinal tract[2] According to the National Cancer Data Base (NCDB) in the United States, the incidence of ocular, and mucosal melanoma were 5.5% and 1.3%, respectively. Intriguingly, the reported incidence of mucosal melanoma varies from 0.2% to 10.0% depending on the ethnic and geographic differences [3-5]. In previous studies comparing melanoma patient cohort cohort, age-adjusted incidence of cutaneous melanoma was highest in non-hispanic whites, and visceral/cutaneous melanoma ratio was much higher for black individuals[6,7]. The proportion of mucosal melanoma seems to be higher in Asian ethnicity when compared with that in Caucasians [8-10]. Moreover, Asian groups showed the lower age-adjusted incidence rate of ocular melanoma compare to caucasian patients [11,12]. Such variations in ethnicity may reflect distinctive pathophysiology or tumorigenesis for each subtypes of melanoma.

The differential distribution of genetic alterations in *BRAF*, *NRAS *and *KIT *among melanoma subtypes according to anatomic sites and sun exposure strongly implicated different molecular pathways involved in tumorigenesis for each subtype (Antonescu *et al*, 2007). *KIT *mutations were detected in 21% of mucosal melanomas, 11% of acral melanomas, and 16.7% of melanomas arising in chronically sun-damaged skin as indicated by the presence of solar elastosis (Antonescu *et al*, 2007). In another recent study, *KIT *mutations were identified in 23% of acral melanomas, 15.6% of mucosal melanomas, 1.7% of cutaneous melanomas, and none in choroidal melanomas[13]. Mucosal melanomas pursued more aggressive natural course and poorer prognosis than other subsets of melanomas[14]. Nevertheless, owing to the rarity and heterogeneity of the disease entity, clinical characteristics and optimal therapeutic strategies have not been extensively defined yet. Given the distinct genetic aberrations for mucosal melanoma, there is an urgent need to redefine the clinical features and prognosis of this subtype of melanoma. Most of the previous reports on mucosal melanomas are focused on head and neck melanomas rather than melanomas arising from mucosal membrane at various anatomic sites[3,14-16]. Hence, we undertook this multicenter retrospective analysis to define clinical characteristics of mucosal melanomas and to devise a prognostic model which can effectively identify different risk groups based on initial clinical variables.

## Methods

### Patients

Between June 1992 and May 2005, a total of 141 patients were newly diagnosed with noncutaneous melanoma at 4 institutions in Korea. The criteria for case inclusion were as following: (1) pathologically confirmed diagnosis of melanoma; (2) complete set of clinical information which was defined by patient demographics, primary tumor site, stage, treatment record, and vital status. Of the 141 patients, 129 patients (91.5%) were histologically confirmed of noncutaneous melanoma with complete set of clinical data available for review. All patients' stage was reclassified using the 2002 American Joint Committee on Cancer (AJCC) melanoma TMN staging classification[17] Mucosal melanomas were defined as tumors arising from mucous membrane such as head and neck, female genital tract, anorectum, and urinary tract. Ocular melanomas were defined as tumors involving the conjunctiva and uvea (arising in iris, ciliary body, and choroid) in this study. This retrospective study was reviewed and approved by the Samsung Medical Center institutional review board (Seoul, Korea).

### Statistics

The primary end point was overall survival (OS), which was calculated using Kaplan-Meier method. OS was defined from the date of surgery to date of death related to the disease or complication. Survival rates were compared for statistical differences using log-rank test. OS was assessed with respect to following factors: age, sex, Eastern Cooperative Oncology Group (ECOG) performance status, presenting symptom, primary site, tumor burden, and resection margin. Multivariate analysis was performed using stepwise Cox proportional hazards regression modeling. P values less than 0.05 were considered statistically significant and all P values corresponded to two-sided significance tests. The latter was performed by Cox's proportional hazard regression model. Because of different characteristics between mucosal and ocular melanoma, we performed analysis separately for those two distinct subsets of patients.

## Results

One-hundred twenty nine patients were included in the analysis. The clinical characteristics are provided in Table 1. After that, we further analyzed survival outcome for those who had received curative resection (R0 and R1 resection) as shown in Table 2. The median age was 61 years with a range of 26 to 95 years and male proportion was 40.3%. The median longest diameter of the primary tumor was 25 mm with a range from 2 to 135 mm. Noncutaneous melanoma cases were further divided into ocular melanoma (n = 14) and mucosal melanoma (n = 115).

**Table 1:** Clinical characteristics of all the 129 patients.

	Mucosal melanoma (n = 115), N (%)							Ocular melanoma (n = 14) N (%)
		
	Total	Anorectal	Genitourinary	Upper GI	Nasal	Oral	Maxillary sinus	
**No.**	**115**	**39 (30.2)**	**21 (16.3)**	**6 (4.7%)**	**30 (23.3)**	**14 (10.9)**	**5 (3.9)**	**14 (10.9)**
**Male (%)**	**45 (39.1)**	14 (35.9)	1 (4.8)	3 (50.0)	17 (56.7)	7 (50.0)	3 (60.0)	7 (50.0)
**Age (years)**								
Median	**61**	62	59	61	63	55	62	64
Range	**26-95**	31-80	26-95	43-72	39-85	33-74	35-75	28-79
**ECOG PS**								
0-1	**113 (98.2)**	38(97.4)	20(95.2)	6(100.0)	30(100.0)	14(100.0)	5(100.0)	13(92.9)
≥ 2	**2(1.8)**	1(2.6)	1(4.8)	0(0.0)	0(0.0)	0(0.0)	0(0.0)	1(7.1)
**Presenting symptom**								
Palpable mass	**70(60.9)**	24(61.5)	16(76.2)	0(0.0)	15(50.0)	13(92.9)	2(40.0)	8(57.1)
Bleeding	**24(20.9)**	10(25.6)	4(19.0)	1(16.7)	7(23.3)	0(0.0)	2(40.0)	0(0.0)
Obstruction	**10(8.7)**	1(2.6)	0(0.0)	0(0.0)	7(23.3)	1(7.1)	1(20.0)	0(0.0)
GI symptom	**9(7.8)**	4(10.3)	0(0.0)	5(83.3)	0(0.0)	0(0.0)	0(0.0)	0(0.0)
Neurologic symptom	**2(1.7)**	0(0.0)	1(4.8)	0(0.0)	1(3.3)	0(0.0)	0(0.0)	6(42.9)
**Size (n = 101, mm)**								
Median (n)	**20**	34 (n = 33)	18.5(n = 14)	32.5(n = 6)	30 (n = 22)	22(n = 10)	15 (n = 5)	12 (n = 11)
Range	**2-135**	2-135	4-75	5-60	11-72	5-40	4-35	4-25
**Lymph node**								
N0	**78 (67.8)**	24(61.5)	14(66.7)	3(50.0)	26(86.7)	7(50.0)	4(80.0)	14(100.0)
N1	**20(17.4)**	7(17.9)	5(23.8)	0(0.0)	3(10.0)	5(35.7)	0(0.0)	0(0.0)
N2	**5(4.3)**	2(5.1)	0(0.0)	1(16.7)	1(3.3)	1(7.1)	0(0.0)	0(0.0)
N3	**12(10.4)**	6(15.4)	2(9.5)	2(33.3)	0(0.0)	1(7.1)	1(20.0)	0(0.0)
**Metastatic site**								
None	**95(82.6)**	32(82.0)	18(85.7)	1(16.7)	28(93.3)	12(85.7)	4(80.0)	13(92.9)
Liver	**10(8.7)**	4(10.3)	3(14.3)	2(33.3)	1(3.3)	0(0.0)	0(0.0)	0(0.0)
Lung	**7(6.1)**	1(2.6)	0(0.0)	3(50.0)	0(0.0)	2(14.3)	1(20.0)	1(7.1)
Peritoneum	**1(0.8)**	1(2.6)	0(0.0)	0(0.0)	0(0.0)	0(0.0)	0(0.0)	0(0.0)
Adrenal	**1(0.8)**	0	0(0.0)	0(0.0)	1(3.3)	0(0.0)	0(0.0)	0(0.0)
Bone	**1(0.8)**	1(2.6)	0(0.0)	0(0.0)	0(0.0)	0(0.0)	0(0.0)	0(0.0)

*GI: gastrointestinal

**Table 2:** Summary of therapeutic and survival data in patients who received curative resection.

	Mucosal melanoma (n = 87), N (%)							Ocular melanoma N (%)
		
	Total	Anorectum	Genitourinary	Gastrointestinal	Nasal	Oral	Maxillary sinus	
**No.**	**87**	**31**	**17**	**1**	**23**	**11**	**4**	**13**
R1 resection	**25**	2(6.5)	5(29.4)	0	12(52.2)	5(45.5)	1(25.0)	5(38.5)
R0 resection	**62**	29(93.5)	12(70.6)	1(100)	11(47.8)	6(54.5)	3(75.0)	8(61.5)
**Adjuvant treatment**								
No	**53**	20(64.5)	8(47.1)	1(100)	18(78.3)	4(36.4)	2(50.0)	7(53.8)
Chemotherapy	**22**	10(32.3)	6(35.3)	0	0(0.0)	4(36.4)	0	2(15.4)
IFN	14	5	4	0	0(0.0)	3	0	2
Cytotoxic	8	5	2	0	0(0.0)	1	0	0
Radiotherapy only	**14**	1(3.2)	3(17.6)	0	5(21.7)	3(27.2)	2(50.0)	4(30.8)
**Relapse after resection**								
None	**36**	11(35.5)	7(41.2)	0	12(52.2)	5(45.4)	1(25.0)	6(46.2)
Local relapse	**21**	7(22.6)	1(5.9)	0	8(34.8)	4(36.4)	1(25.0)	4(30.8)
Systemic	**30**	13(41.9)	9(52.9)	1(100)	3(13.0)	2(18.2)	2(50.0)	3(23.0)
**Palliative treatment after recurrence (total n = 51)**								
Chemotherapy only	**9 (17.6)**	3	2	0	3	1	0	3(42.9)
Radiotherapy only	**10 (19.6)**	3	2	0	2	3	0	1(14.3)
Resection	**6 (11.7)**	2	1	1	0	1	1	0
**Current status**								
No evidence of disease	**31 (35.6)**	12(38.7)	7(41.2)	0	7(30.4)	5(45.5)	0(0.0)	5(38.5)
Alive with disease	**6 (6.9)**	1(3.2)	1(5.9)	0	2(8.7)	1(9.0)	1(25.0)	2(15.3)
Dead of disease	**50 (57.5)**	18(58.1)	9(52.9)	1(100)	14(60.9)	5(45.5)	3(75.0)	6(46.2)
**Overall Survival (%)**								
2 years	**59.7**	61.3	68.4	-	46.9	53.0	75.0	73.3
5 years	**31.9**	30.6	39.1	-	25.1	39.8	-	-
**Median survival months (95% CI)**	**34.6** **(24.5-44.7)**	28.6 (18.2-38.9)	43.9 (29.6-58.2)	7.5 (NA)	23.4 (7.4-8.9)	38.9 (13.4-64.4)	24.4 (0-49.3)	51.2 (NA)

### Mucosal melanoma

#### Patient Characteristics

Anorectum was the most common anatomic site (n = 39, 30.2%) which was followed by nasal cavity (n = 30, 23.3%), genitourinary (n = 21, 16.3%), oral cavity (n = 14, 10.9%), upper gastrointestinal tract (n = 6, 4.7%) and maxillary sinus (n = 5, 3.9%) in the order of frequency. The age distributions were similar among different anatomic locations. In terms of gender, seventy seven patients (59.7%) were wemen, and fifty two (40.3%) were men, with a female-to-male ratio of 1.48:1. For those with genitourinary and anorectual melanoma, especially more female dominant patients were noticed (95.2% and 64.1%, respectively). Palpable mass was the most common initial symptom, followed by bleeding, and obstructive symptoms. However, patients with upper gastrointestinal (GI) tract mucosal melanoma presented often with GI symptoms, such as dyspepsia, dysphagia, and epigastric discomfort. Of note, 83.3% of melanomas arising from upper gastrointestinal tract presented with initially disseminated disease, which was much higher than the rate of metastatic disease in all cases (16.3%). In all, the most common site of metastasis was liver (n = 10, 7.8% of all cases), followed by lung.

#### Treatment modalities and outcome

Total 87 out of 115 patients (75.6%) received curative intended surgery, consisted of 62 R0 resected and 25 R1 resected patients. After that, 36 (41.4%) patients received postoperative adjuvant treatment, 14 were treated with radiotherapy, 22 had interferon-2bα (n = 14) or dacarbazine-based (n = 8) chemotherapy. In terms of relapse pattern, head and neck sites has a tendency to recur locally, whereas rectal and genitourinary lesions recurred systemically.

For anorectal melanomas, 31 patients underwent wide excision with regional lymph node dissection and 10 patients with primary resection received postoperative interferon-2bα (n = 5) or dacarbazine-based chemotherapy (n = 5) (Table 2). Of the 31 anorectal melanoma patients with curative resection, 20 (64.5%) patients developed recurrent disease with 7 local relapse and 13 systemic relapses. The median survival time for anorectal melanomas was 28.6 months (95% CI, 18.2, 38.9 months). For genitourinary melanoma (n = 21), 17 (81.0%) patients underwent radical cancer surgery and nine patients received postoperative treatment (interferon-2bα, n = 4; chemotherapy, n = 2; radiotherapy, n = 3). Median survival time was 43.9 months which is longer than that for anorectal melanoma, and approximately half of the curatively resected genitourinary melanoma patients developed systemic relapses. Melanomas arising from upper gastrointestinal tract (n = 6) pursued the most aggressive clinical course with median survival time of only 7.5 months. Five out of six gastrointestinal melanoma patients presented with initially metastatic disease. Of 30 nasal melanoma cases, 23 patients underwent radical cancer surgery with curative intent and 11 patients had R0 resections. The median survival time for curatively resected nasal melanoma patients was only 23.4 months and 14 patients died of the disease.

#### Survival and prognostic factors for survival

**Figure 1:**
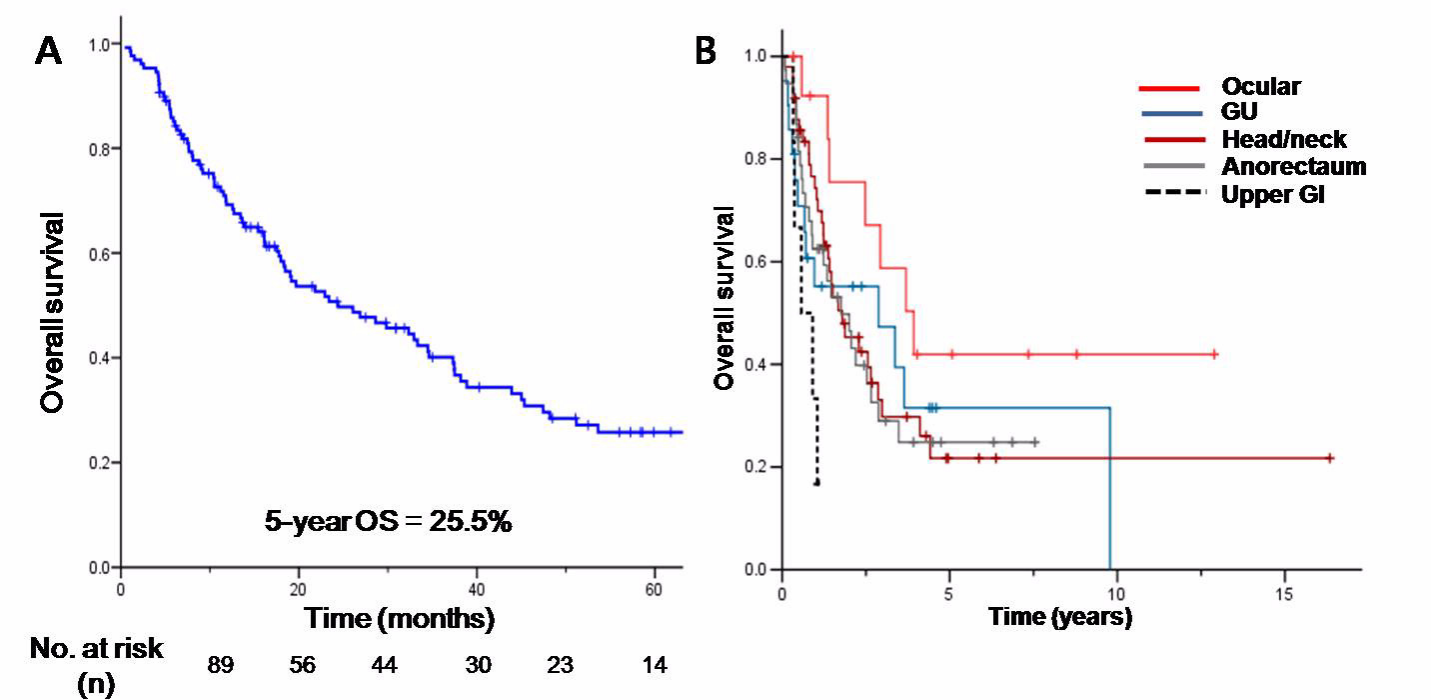
**Survival of all noncutaneous melanoma patients (A) and (B) according to primary sites**.

After 64.5 (range 4.3-213.0) months of median follow-up, an estimated 2 and 5-year OS rates in 129 patients were 50.7% and 25.5%, respectively (Figure 1A). At the time of analysis, 62.8% (n = 81) of patients were dead. The median overall survival for all patients was 24.4 months (95% CI 13.2-35.5). Interestingly, survival rate varied according to different primary anatomic sites (Figure 1B).

Because of different characteristics of ocular melanoma, we further analyzed of prognostic factors for the curatively resected 115 mucosal melanoma patients. At univariate analysis, patients with age ≤ 50 years lived longer than those older than 50 years with statistical significance (3 year OS, 70.1% *vs *33.3%, respectively; *P *= .003) as shown in Table 3. For the resection margin, patients with R0 resection margin demonstrated significantly prolonged survival time compared to those with R1 resection (median OS; 37.5 *vs *16.1 months, respectively, *P *= 0.001). Patients with oral and genitourinary tract melanoma demonstrated slightly better tendency of survival than the others (median OS; 43.9 vs 26.8 months, *P *= 0.26). In addition, patients with positive regional LN stage 0-1 demonstrated slightly poorer survival than patients with LN 2-3 (median OS 32.9 *vs *10.5 months, *P *= .017). However, patient's gender, and additional adjuvant therapy had no statistically significant effect on survival outcomes. We could not analyze about the tumor thickness because of incomplete data.

**Table 3:** Prognostic Factors of Survival for 87 mucosal melanoma patients who had received curative resection (R0 and R1 resection).

	Univariate	Multivariate analyses	
	
Parameters	*P *value	Relative Risk (exp. B)	*P *value
Age (≤ 50 years *vs *> 50 years)	0.003	2.88 (1.32-5.39)	0.008
Sex (female *vs *male)	0.43	1.21 (0.66-2.23)	0.54
Adjuvant treatment (done *vs *not done)	0.76	1.02 (0.54-1.94)	0.94
Primary site (oral and genitourinary *vs *others)	0.26	1.63 (0.81-3.29)	0.17
Lymph nodes (N0-1 *vs *N2-3)	0.17	1.91 (0.51-7.19)	0.34
Complete resectability (RO *vs *R1)	0.001	2.93 (1.58-6.26)	0.001

**Figure 2:**
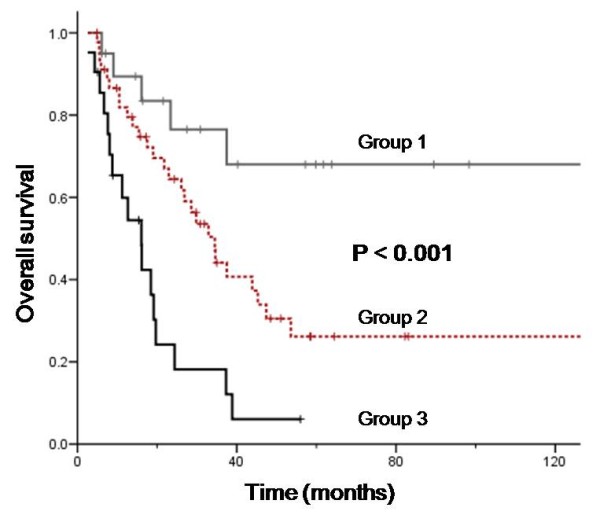
**Survival according risk group as defined by prognostic models**.

We performed multivariate analysis to evaluate the independent prognostic factors of survival for 87 mucosal melanoma patients who had received curative resection (R0 and R1 resection) as shown in Table 3. Clinical parameters predicting poor survival outcome that were included in the multivariate analysis were as follows: age (> 50 years *vs *= 50 years), gender (female *vs *male), primary site (oral and genitoruinary *vs *the other mucosal melanomas), lymph node status (N0-1 *vs *N2-3), complete resectability (R0 *vs *R1 resection). The entered Cox regression model was used to delineate independent prognostic factors. Prognostic factors for survival were primary site (*P *= .03; relative risk [RR], 2.4; 95% CI, 1.1, 5.4), complete resectability (*P *= .001; RR, 2.92; 95% CI, 1.58-6.26), and age (*P *= .008; RR, 2.88; 95% CI, 1.32-5.39). The prognostic grouping of the 87 patients was performed according to the following criteria: Group 1 (n = 20), no adverse factor; Group 2 (n = 46), one adverse factors; and Group 3 (n = 21), two adverse factors. The survival curves according to the prognostic index are demonstrated in Figure 2. The prognostic model separated patients into three risk groups with different survival outcomes (*P *< .001). The 2-year OS rates for Group 1, 2, and 3 were 76.5, 64.4, and 24.2%, respectively (Table 4). Group 3 patients had seven folds (HR 7.29, 95% CI 2.64-21.11) increased risk of death compared to Group 1.

**Table 4:** Survival and relative risk of death according to risk group as defined by prognostic model.

Risk group	No. of factors *	No. of patients (%)	% of 2-Year OS (SE)	RR (95% CI)
**Group 1**	0	20 (23.0)	76.5 (10.4)	1
**Group 2**	1	46 (52.9)	64.4 (7.4)	2.94 (1.20-7.63)
**Group 3**	2	21 (24.1)	24.2 (10.3)	7.29 (2.64-21.11)

### Ocular melanoma

For 14 ocular melanoma, 11 were choroidal melanoma, and 3 were conjunctival melanoma patients. Age and gender distribution was similar with other mucosal melanoma patients. Almost all the patients (92.9%) presented without distant metastasis, and only 1 of 14 cases presented with metastatic disease to lung (for uveal melanoma patient). In terms of treatment, all ocular melanoma patients except one underwent resection with curative intent (8 R0 resection and 5 R1 resection). Survival outcome was much better than mucosal melanoma, which demonstrated 73.3% of 2 year OS and 51.2 months of median OS.

## Discussion

To provide a more complete clinical picture of this uncommon disease entity and ultimately deduce the risk-adapted therapeutic strategies, we analyzed the largest series of noncutaneous melanomas in terms of clinical features and prognosis. In this study, we depicted distinctive clinical features of the rare disease entity and categorized heterogeneous subsets of noncutaneous melanoma into two groups: ocular and nonocular mucosal melanoma. Moreover, systemic analyses of prognostic factors reflecting poor survival have not been reported in noncutaneous melanoma patients. Hence, we attempted to devise a prognostic model incorporating readily available clinical variables which may assist clinicians for risk stratification.

In this study, the median overall survival for all patients and mucosal melanoma patients were 24.4 months (95% CI 13.2-35.5) and 34.6 months (95% CI 24.5-44.7), which is in accordance with previous studies[18,19]. Based on those findings, we analyzed significant prognostic factors of OS for mucosal melanoma patients who had received curative resection. The significant poor prognostic factors were microscopic residual disease (*P *= .001; RR, 2.93; 95% CI 1.58-6.26), and age > 50 years (*P *= .008; RR, 2.88; 95% CI, 132-5.39) (Table 3). These findings are very consistent with previous study for mucosal melanoma [15]. In the multivariate analysis, age greater than 60 years was one of the poor prognostic factors for survival. Contrary to cutaneous melanoma, achieving extensive resection is very difficult due to anatomical location or cosmetic concerns, and performing radical lymphadenectomy is much more challenging. Based on those results, more attention is required for achieving R0 resection for mucosal melanoma patients. In addition, even after the curative intended resection, patients with microscopically residual resection should be offered of more aggressive therapeutic strategy such as postoperative chemotherapy ± radiotherapy. Due to the inherent bias for retrospective study in nature and the limited sample size, this result should be prospectively validated before clinical application.

Importantly, the survival outcome seems to differ according to the primary site of nonocular mucosal melanoma. For instance, 5 of 6 gastrointestinal melanomas presented with disseminated disease and pursued a rapidly deteriorating natural course with median survival time of 7.5 months. There are several anecdotal case reports on primary gastric or esophageal melanoma which reported poor survival following resection in accordance with our observation [20-22]. In contrast, ocular melanomas exceptionally pursued a favorable clinical course with median survival of 51.2 months. Most of the ocular melanoma patients (13 of 14, 93%) presented with localized disease of whom eight achieved R0 resection. One of the plausible explanations for such significant difference in prognosis would owe to the anatomic location. For instance, ocular melanoma may be more noticeable and symptomatic than gastric melanoma; thus, over 90% of the ocular melanoma patients are diagnosed at localized stage. As shown in Figure 1B, genitourinary, anorectal and head and neck melanomas demonstrated relatively similar survival pattern.

In all, of the 100 noncutaneous melanoma patients who underwent surgical resection with curative intent, 70 patients achieved complete R0 resection and 58 developed recurrence (43% local relapse, 57% systemic relapse). Of the 51 recurred patients, only 6 patients underwent re-resection and half of the patients did not receive palliative treatment. Considering the high rate of systemic relapse following curative resection observed in our study along with previous reports [14,23], an effective postoperative systemic treatment is urgently needed. The role of adjuvant treatment in mucosal melanoma has not been extensively studied compared to cutaneous melanoma probably owing to the rarity of disease. Several studies on adjuvant treatment using chemotherapeutic or immunotherapeutic agents, the results have been disappointing[4,24-27]. In line with these studies, our retrospective analysis on 100 noncutaneous melanoma patients with surgical resection showed no significant difference in survival associated with adjuvant chemotherapy (median survival; 45.3 months *vs *34.5 months, adjuvant therapy *vs *observation, respectively, *P *= .085). In subgroup analysis, however, there was a trend toward better survival in anorectal and genitourinary mucosal melanoma patients who received postoperative chemotherapy (median OS 45.3 months vs 26.1 months, respectively, *P *= .20). Therefore, the role of adjuvant chemotherapy or immunotherapy should be actively sought in noncutaneous melanoma in the context of clinical trial.

A potential breakthrough in the management of mucosal melanoma has been recently suggested by the observation of relatively high incidence of *KIT *mutations in mucosal melanomas [28-30]. Excitingly, a major response has been reported following imatinib treatment in primary anal melanoma patient harboring a seven-codon duplication in exon 11 of *KIT *[31]. Other reports also support the use of specific kinase inhibitors such as imatinib or dasatinib in melanoma patients with activating *KIT *mutation [30,32]. Given the high incidence of KIT mutations in mucosal melanoma, we plan to conduct a phase II clinical trial with *KIT *targeting small molecule in this subset of patients.

## Conclusion

This is the first study to comprehensively review and describe the clinical features and prognosis of noncutaneous melanoma. Nonocular mucosal melanoma pursued an aggressive clinical course and high rate of systemic relapse following curative resection and thus, more aggressive postoperative treatment should be considered for the patients with poor risk factors. Moreover, further validation and postoperative study with different strategy is warranted in the future.

## Competing interests

The authors declare that they have no competing interests.

## Authors' contributions

HSK drafted the manuscript. HSK, EKK, HJJ, SYO, DHL, KWP, JHK, KMK and SIL collected the data and performed the statistical analysis. HSK, SYO, DHL, KWP, JHK, SIL, DHL, and JL followed the patients. DHL and JL designed the study and helped with the manuscript. All authors read and approved the final manuscript.

## Pre-publication history

The pre-publication history for this paper can be accessed here:

http://www.biomedcentral.com/1471-2407/10/167/prepub
